# Impact of chronic GLP-1 RA and SGLT-2I therapy on in-hospital outcome of diabetic patients with acute myocardial infarction

**DOI:** 10.1186/s12933-023-01758-y

**Published:** 2023-02-06

**Authors:** Filippo Trombara, Nicola Cosentino, Alice Bonomi, Monica Ludergnani, Paolo Poggio, Luigia Gionti, Marta Baviera, Pierluca Colacioppo, Maria Carla Roncaglioni, Olivia Leoni, Francesco Bortolan, Piergiuseppe Agostoni, Stefano Genovese, Giancarlo Marenzi

**Affiliations:** 1 Centro Cardiologico Monzino, I.R.C.C.S, Via Parea 4, 20138 Milan, Italy; 2 Cardiovascular Section, Department of Clinical Sciences and Community Health, University of Milan, Milan, Italy; 3 Laboratory of Cardiovascular Prevention, Istituto Di Ricerche Farmacologiche Mario Negri IRCCS, Milan, Italy; 4 Regional Epidemiological Observatory, Lombardy Region, Milan, Italy

**Keywords:** Glucagon-like peptide-1 receptor agonists, Sodium glucose cotransporter-2 inhibitors, Diabetes mellitus, Acute myocardial infarction, In-hospital outcome

## Abstract

**Background:**

Glucagon-like peptide-1 receptor agonists (GLP-1 RA) and sodium glucose cotransporter-2 inhibitors (SGLT-2i) demonstrated cardiovascular and renal protection. Whether their benefits occur also during hospitalization for acute myocardial infarction (AMI) in patients with diabetes mellitus (DM) is not known. We evaluated in-hospital outcomes of patients hospitalized with AMI according to their chronic use of GLP-1 RA and/or SGLT-2i.

**Methods:**

Using the health administrative databases of Lombardy, patients hospitalized with AMI from 2010 to 2019 were included. They were stratified according to DM status, then grouped into three cohorts using a propensity score matching: non-DM patients; DM patients treated with GLP-1 RA and/or SGLT-2i; DM patients not treated with GLP-1 RA/SGLT-2i. The primary endpoint of the study was the composite of in-hospital mortality, acute heart failure, and acute kidney injury requiring renal replacement therapy.

**Results:**

We identified 146,798 patients hospitalized with AMI (mean age 71 ± 13 years, 34% females, 47% STEMI; 26% with DM). After matching, 3,090 AMI patients (1030 in each group) were included in the analysis. Overall, the primary endpoint rate was 16% (n = 502) and progressively increased from non-DM patients to DM patients treated with and without GLP-1 RA/SGLT-2i (13%, 16%, and 20%, respectively; P < 0.0001). Compared with non-DM patients, DM patients with GLP-1 RA/SGLT-2i had a 30% higher risk of the primary endpoint, while those not treated with GLP-1 RA/SGLT-2i had a 60% higher risk (P < 0.0001).

**Conclusion:**

Chronic therapy with GLP-1 RA and/or SGLT-2i has a favorable impact on the clinical outcome of DM patients hospitalized with AMI.

**Supplementary Information:**

The online version contains supplementary material available at 10.1186/s12933-023-01758-y.

## Introduction

Diabetes mellitus (DM) is a relevant risk factor and frequent comorbidity in patients hospitalized with acute myocardial infarction (AMI), with a prevalence of 20–30% [1–3]. Although current treatments have considerably improved survival in both DM and non-DM patients with AMI, the presence of DM still carries a higher risk of in-hospital mortality and major cardiovascular complications, doubling the case fatality rate [4–7]. This is likely due to multifactorial causes, including a higher number of comorbidities and burden of coronary artery disease, as well as an increase in inflammation and prothrombotic state [7]. Moreover, it has been recently demonstrated that the higher in-hospital mortality in DM patients with AMI is mainly driven by their higher rate of cardiac and renal dysfunction [8]. This suggests that an improvement in hospital outcomes in the DM population may be achieved through the early implementation of cardio-protective and renal-protective therapies, in addition to standard of care.

Long-term treatment with two classes of drugs for DM—glucagon-like peptide-1 receptor agonists (GLP-1 RA) and sodium glucose cotransporter-2 inhibitors (SGLT-2i)—demonstrated cardiovascular and renal protection in addition to their glucose-lowering effect [9–13]. Whether the benefit of these drugs on cardio-renal protection can also be observed in the acute phase of AMI, with a favorable impact on hospital outcomes, has not been investigated yet. Few preliminary data seem to support this hypothesis. Indeed, GLP-1 RA therapy was shown to reduce myocardial infarct size in animal models [14] and reperfusion injury in patients with ST-segment elevation myocardial infarction (STEMI) [15]. More recently, SGLT-2i were found to preserve cardiac contractile function during myocardial ischemia–reperfusion injury in an animal model [16]. They were also shown to prevent kidney function decline in DM patients after AMI [17]. While waiting for the results of ongoing randomized clinical trials focusing on the protective role of these drugs in AMI patients [18], the evaluation of in-hospital outcomes in DM patients, chronically treated with these anti-hyperglycemic agents at the time of hospitalization with AMI, represents a good opportunity to explore their potential role in this acute clinical setting.

In this study, we analyzed administrative data from Lombardy, the most populous Italian region, with the aim to evaluate in-hospital outcomes in patients hospitalized with AMI according to their DM status and their anti-hyperglycemic therapy at the time of hospitalization. In particular, we focused our analysis on the in-hospital prognosis of patients on chronic GLP-1 RA and/or SGLT-2i therapy.

## Methods

### Data source

Our study used connected administrative health databases of the Lombardy region, Italy, which include population registries with demographic data of all residents and detailed information on drug prescriptions, hospital records, and medical exemptions. Data are available for about 10 million inhabitants since 2000. Healthcare in Italy is publicly funded for all residents, irrespective of social class or employment, and everyone is assigned a personal identification number kept in the National Civil Registration System. All residents are assisted by general practitioners under the national health system. The pharmaceutical prescription database contains the medication name and Anatomic Therapeutic Chemical classification (ATC) code, quantity, and date of the dispensation of drugs reimbursed by the national health system. The hospital databases contain information on the date of admission, discharge, death, primary diagnosis, and up to five co-existing clinical conditions and procedures received.

The diagnoses are uniformly coded according to the 9^th^ International Code of Diseases (ICD-9-CM) and standardized in all Italian hospitals. They are compiled by the hospital specialists directly in charge of the patients upon their discharge, and are validated by hospitals against detailed clinical-instrumental data, as they determine reimbursement from the national health system. A unique identification code allows the linkage of all databases. To ensure privacy, each identification code was automatically converted into an anonymous code before we received the dataset. In Italy, studies using retrospective aggregated-anonymous data from administrative databases do not require Ethics Committee approval nor notification. The data underlying this article were provided by the Lombardy region by permission and cannot be shared without permission of the Lombardy region.

### Study cohorts

All patients with a hospitalization due to AMI as a primary diagnosis (both STEMI and non-ST-elevation myocardial infarction [ICD-9-CM codes 410.x]) from January 1, 2010, through December 31, 2019, were initially screened. We included in this study patients since 2010 because GLP-1 RA and SGLT-2i were available in the Italian market from 2010 and 2015, respectively. Patients were stratified according to their DM status at the time of hospitalization. Diabetes mellitus was defined as chronic exposure to anti-hyperglycemic agents (at least two prescriptions of ATC code A10* within the year preceding index hospitalization). Patients with DM were further stratified according to GLP-1 RA/SGLT-2i treatment for at least 3 months before index hospitalization. When patients were transferred between hospitals, we evaluated the complete episode of care.

### Study endpoints

The primary endpoint of the study was the composite of in-hospital mortality, acute heart failure, and acute kidney injury requiring renal replacement therapy (RRT). To avoid interference, each patient could only account for one event classification. Each component of the primary endpoint evaluated separately was considered as the secondary endpoint. The two non-fatal components of the primary endpoint were retrieved from the clinical conditions (acute heart failure) and procedures (RRT) coded in hospital records.

### Statistical analysis

A propensity score matching was used to reduce confounding due to imbalance in study covariates. The score was used to match the following three cohorts: non-DM patients; DM patients treated with GLP-1 RA and/or SGLT-2i as part of their therapy; and DM patients not treated with GLP-1 RA and/or SGLT-2i. The three groups were matched in a 1:1:1 ratio using all variables included in Table 1 (age, gender, year of index hospitalization, major comorbidities, AMI type, cardiovascular medications before hospital admission, and percutaneous coronary intervention during hospitalization). Anti-hyperglycemic therapy before hospitalization, not present by definition in patients without DM, was excluded from the propensity score matching.

**Table 1:** Clinical characteristics of patients hospitalized with acute myocardial infarction from 2010 to 2019 prior to propensity score matching

	Non-DM pts (n = 108,815)	DM pts treated with GLP-1 RA/SGLT-2i (n = 1030)	DM pts not treated with GLP-1 RA/SGLT-2i (n = 36,953)	*p value*	*p value*^a^
Age (years)	70 ± 14	66 ± 9	75 ± 11	< 0.0001	< 0.0001
Female gender, n (%)	36,537 (34%)	279 (27%)	13,612 (37%)	< 0.0001	< 0.0001
Year of hospitalization	2015 (2013–2017)	2017 (2016–2019)	2014 (2013–2017)	< 0.0001	< 0.001
Prior MI, n (%)	17,764 (16%)	334 (32%)	11,127 (30%)	< 0.0001	0.12
STEMI as admission diagnosis, n (%)	54,329 (50%)	351 (34%)	13,621 (37%)	< 0.0001	0.06
Comorbidities^b^					
Arterial hypertension, n (%)	30,571 (28%)	486 (47%)	16,702 (45%)	< 0.0001	0.23
Chronic IHD, n (%)	27,772 (25%)	490 (47%)	13,259 (36%)	< 0.0001	< 0.0001
CKD, n (%)	4494 (4%)	62 (6%)	5017 (14%)	< 0.0001	< 0.0001
COPD, n (%)	3706 (3%)	52 (5%)	2600 (7%)	< 0.0001	0.01
Cancer, n (%)	10,104 (9%)	88 (9%)	4315 (12%)	< 0.0001	0.002
Atrial fibrillation, n (%)	5929 (5%)	57 (5%)	3136 (8%)	< 0.0001	0.0007
Chronic heart failure, n (%)	570 (0.5%)	14 (1%)	427 (1%)	< 0.0001	0.55
Number of comorbidities				< 0.0001	0.01
0, n (%)	51,217 (46%)	255 (25%)	10,258 (28%)		
1, n (%)	38,289 (35%)	425 (41%)	13,865 (37%)		
2, n (%)	14,375 (13%)	248 (24%)	8358 (23%)		
3, n (%)	3837 (4%)	89 (9%)	3260 (9%)		
> 3, n (%)	1097 (1%)	15 (1%)	1212 (3%)		
Medications before hospitalization					
ACE-I/ARBs, n (%)	51,877 (48%)	777 (75%)	26,426 (71%)	< 0.0001	0.008
Antihypertensive drugs, n (%)	74,782 (697%)	914 (89%)	32,661 (88%)	< 0.0001	0.86
Beta-blockers, n (%)	33,789 (31%)	595 (58%)	19,181 (52%)	< 0.0001	0.0003
Lipid lowering drugs, n (%)	34,162 (31%)	828 (80%)	22,458 (61%)	< 0.0001	< 0.0001
Antiplatelet drugs, n (%)	40,204 (37%)	626 (61%)	23,027 (62%)	< 0.0001	0.2792
Anticoagulant drugs, n (%)	6205 (6%)	69 (7%)	3515 (9%)	< 0.0001	0.0022
Oral anti-hyperglycemic drugs	–	1030 (100%)	25,814 (70%)	< 0.0001	< 0.0001
Metformin, n (%)	–	836 (81%)	17,931 (49%)	–	< 0.0001
Sulfonylurea, n (%)	–	299 (29%)	10,434 (29%)	–	0.63
DPP-4i, n (%)	–	91 (9%)	2870 (8%)	–	0.24
Insulin, n (%)	–	466 (45%)	12,112 (33%)	–	< 0.0001
Procedures during index hospitalization					
Coronary multivessel disease, n (%)	57,215 (53%)	581 (56%)	16,231 (44%)	< 0.0001	< 0.0001
PCI, n (%)	70,610 (65%)	732 (71%)	20,441 (55%)	< 0.0001	<0 .0001
DES implantation, n (%)	56,427 (52%)	661 (64%)	16,340 (44%)	< 0.0001	< 0.0001
Endpoints					
Primary endpoint, n (%)	18,144 (17%)	166 (16%)	10,722 (29%)	< 0.0001	<0 .0001
In-hospital mortality, n (%)	6323 (6%)	35 (3%)	2714 (7%)	< 0.0001	< 0.0001
Acute heart failure, n (%)	13,247 (12%)	137 (13%)	8443 (23%)	< 0.0001	<0 .0001
AKI requiring RRT, n (%)	781 (1%)	3 (0.3%)	912 (3%)	< 0.0001	< 0.0001

^a^P value refers to the comparison between DM patients treated with and not treated with GLP-1 RA/SGLT-2i

^b^in the previous 10 years

*ACE-I* angiotensin-converting-enzyme inhibitors, *AKI* acute kidney injury, *ARBs* angiotensin II receptor blockers, *CKD*  chronic kidney disease, *COPD* chronic obstructive pulmonary disease, *DES* drug-eluting stent, *DM* diabetes mellitus, *DPP-4i*  dipeptidyl peptidase-4 inhibitors, IHD ischemic heart disease, *MI* myocardial infarction, *PCI* percutaneous coronary intervention, *RRT* renal replacement therapy, *STEMI* ST-elevation myocardial infarction

Continuous variables are presented as mean ± standard deviation while non-normally distributed variables as median and interquartile ranges. The differences between the three study groups were assessed using ANOVA and Mann–Whitney tests, as appropriate. Categorical variables were described using frequencies and percentages, and compared using χ^2^ -test. Differences among the three groups for continuous and categorical variables were assessed by ANOVA and χ2-test, respectively.

Univariate and multivariate logistic regression analyses were performed to estimate odds ratios (OR) and 95% confidence intervals (CI) for each study endpoint. The P-values of interaction between gender and the three study groups were calculated.

A sensitivity analysis having DM patients with AMI and treated with dipeptidyl peptidase-4 inhibitors (DPP-4i) as a comparator cohort was also carried out. This comparison allowed to assess the effects of GLP-1 RA and/or SGLT-2i versus a drug class, i.e. DPP4-i, known to exhibit neutral effects on major cardiovascular and renal outcomes in DM patients [20, 21]. Moreover, GLP-1 RA, SGLT-2i, and DPP-4i are only prescribed by diabetes specialists in Italy.

A two-sided P-value less than 0.05 was required for statistical significance. All the analyses were performed using SAS version 9.4 (SAS Institute, Cary, NC, USA).

## Results

**Fig. 1:**
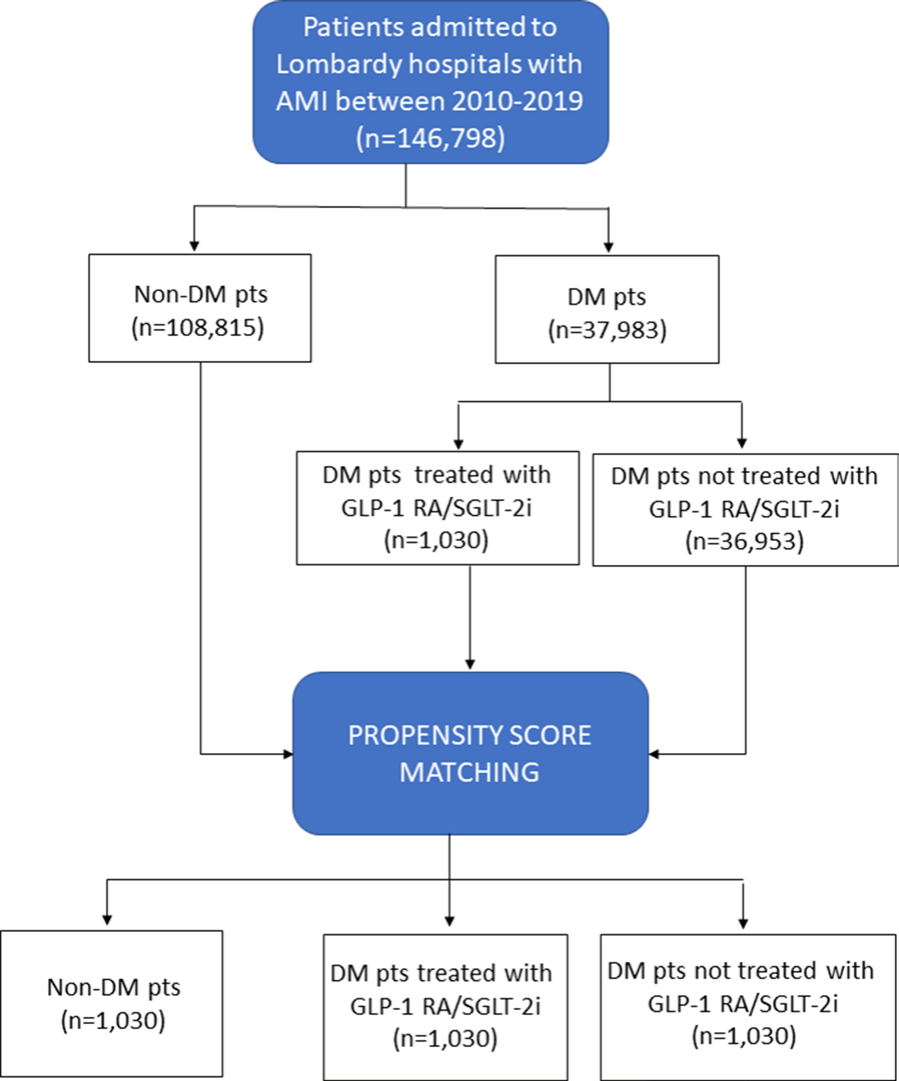
Screening and Selection. Flow chart illustrating study patient screening and selection. *AMI* acute myocardial infarction, *DM* diabetes mellitus, *GLP-1 RA* glucagon-like peptide-1 receptor agonists, *SGLT-2i* sodium glucose cotransporter-2 inhibitors

During the considered study period (2010–2019), 146,798 patients hospitalized with a primary diagnosis of AMI (mean age 71 ± 13 years, 34% females, 47% STEMI; 26% patients with DM) were identified. Of them, 1,030 patients with DM were treated with GLP-1 RA/SGLT-2i (median time of drug administration prior to AMI 19 [6–27] months). Clinical characteristics and pharmacological therapy at hospital admission of the three groups before propensity score matching are reported in Table 1. After propensity score matching, the study population included 3,090 AMI patients (1030 patients in each group) (Fig. 1). Baseline characteristics of study patients after propensity score matching are shown in Table 2. After matching, all variables were well-balanced among groups but anti-hyperglycemic agents.

**Table 2:** Clinical characteristics of the final study population hospitalized from 2010 to 2019 after propensity score matching^a^

	Non-DM pts (n = 1030)	DM pts treated with GLP-1 RA/SGLT-2i (n = 1030)	DM pts not treated with GLP-1 RA/SGLT-2i (n = 1030)	*p value*	*p value*^b^
Age (years)	67 ± 13	66 ± 9	67 ± 11	0.22	0.43
Female gender, n (%)	289 (28%)	279 (27%)	268 (26%)	0.58	0.58
Year of hospitalization	2018 (2016–2019)	2019 (2016–2019)	2018 (2016–2019)	0.34	0.72
Prior MI, n (%)	335 (32%)	333 (32%)	363 (35%)	0.29	0.16
STEMI as admission diagnosis, n (%)	355 (34%)	350 (34%)	347 (34%)	0.93	0.89
Comorbidities^c^					
Arterial hypertension, n (%)	494 (48%)	485 (47%)	488 (47%)	0.92	0.86
Chronic IHD, n (%)	478 (46%)	488 (47%)	495 (48%)	0.75	0.76
CKD, n (%)	59 (6%)	62 (6%)	67 (6%)	0.76	0.65
COPD, n (%)	53 (5%)	52 (5%)	47 (5%)	0.81	0.61
Cancer, n (%)	99 (19%)	88 (8%)	97 (9%)	0.67	0.49
Atrial fibrillation, n (%)	51 (5%)	57 (5%)	60 (6%)	0.67	0.77
Chronic heart failure, n (%)	14 (1%)	14 (1%)	10 (1%)	0.65	0.42
Number of comorbidities				0.16	0.15
0, n (%)	246 (24%)	255 (25%)	231 (22%)		
1, n (%)	442 (43%)	424 (41%)	434 (42%)		
2, n (%)	247 (24%)	247 (24%)	285 (27%)		
3, n (%)	73 (7%)	89 (9%)	61 (6%)		
> 3, n (%)	22 (2%)	15 (1%)	19 (2%)		
Medications before hospitalization					
ACE-I/ARBs, n (%)	772 (75%)	775 (75%)	804 (78%)	0.19	0.13
Beta blockers, n (%)	590 (57%)	595 (58%)	600 (58%)	0.90	0.82
Lipid lowering drugs, n (%)	811 (79%)	826 (80%)	831 (81%)	0.52	0.78
Antiplatelet drugs, n (%)	638 (62%)	624 (61%)	613 (59%)	0.53	0.67
Anticoagulant drugs, n (%)	73 (7%)	69 (7%)	78 (8%)	0.74	0.44
Anti-hyperglycemic drugs	–				
Metformin, n (%)	–	836 (81%)	621 (60%)	–	< 0.0001
Sulfonylurea, n (%)	–	299 (29%)	239 (23%)	–	0.003
DPP-4i, n (%)	–	91 (9%)	112 (11%)	–	0.12
Insulin, n (%)	–	466 (45%)	12,112 (33%)	–	< 0.0001
Procedures during index hospitalization					
Coronary multivessel disease, n (%)	554 (54%)	579 (56%)	581 (56%)	0.41	0.93
PCI, n (%)	724 (70%)	730 (71%)	739 (72%)	0.76	0.66
DES implantation, n (%)	640 (62%)	659 (64%)	650 (63%)	0.68	0.68

^a^Propensity score matching was performed considering all variables included in Table 1, except anti-hyperglycemic drugs

^b^P value refers to the comparison between DM patients treated with and not treated with GLP-1 RA/SGLT-2i

^c^in the previous 10 years

*ACE-I* angiotensin-converting-enzyme inhibitors, *ARBs* angiotensin II receptor blockers, *CKD* chronic kidney disease, *COPD* chronic obstructive pulmonary disease, *DES* drug-eluting stent, *DM* diabetes mellitus, *IHD* ischemic heart disease; *MI* myocardial infarction, *PCI* percutaneous coronary intervention, *STEMI* ST-elevation myocardial infarction

**Fig. 2:**
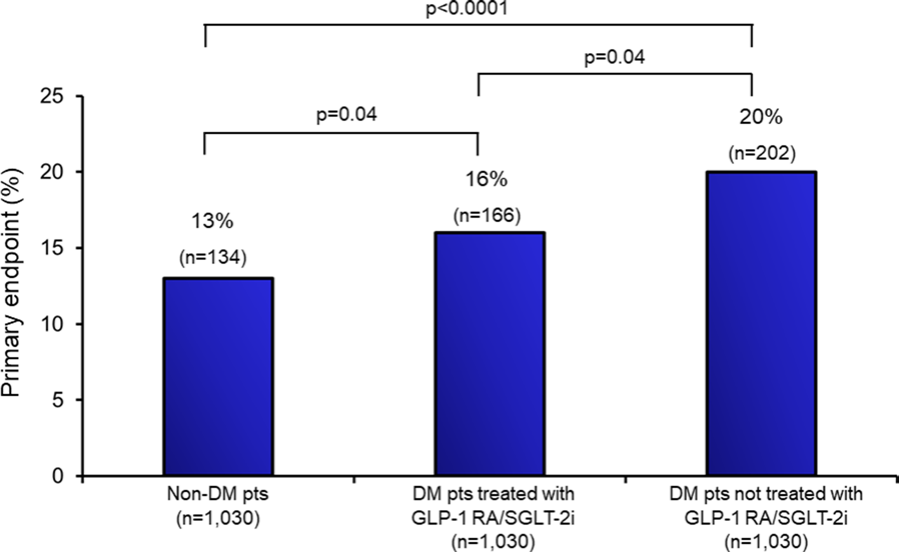
Primary endpoint in the three study groups, after propensity score matching. Composite of in-hospital mortality, acute heart failure, and acute kidney injury requiring renal replacement therapy in patients without diabetes mellitus and in those with diabetes mellitus treated or not with GLP-1 RA/SGLT-2i. *AKI* acute kidney injury, *DM* diabetes mellitus, *GLP-1 RA* glucagon-like peptide-1 receptor agonists, *RRT* renal replacement therapy, *SGLT-2i* sodium glucose cotransporter-2 inhibitors

The primary endpoint rate in the overall (non-matched) population was 20% (n = 29,032), and it was 17%, 16%, and 29%, in non-DM patients, in DM patients treated with GLP-1 RA/SGLT-2i, and in DM patients not treated with GLP-1 RA/SGLT-2i, respectively (Table 1). The primary endpoint rate in the overall matched population was 16% (n = 502). It was higher in patients with DM than in those without (18% vs. 13%; P < 0.0001; OR 1.45 [1.17–1.80]) and it progressively increased going from non-DM patients to DM patients not treated with GLP-1 RA/SGLT-2i (Fig. 2). Notably, the positive clinical effect associated with these two classes of drugs was mainly driven by the reduction in the incidence of acute heart failure.

**Fig. 3:**
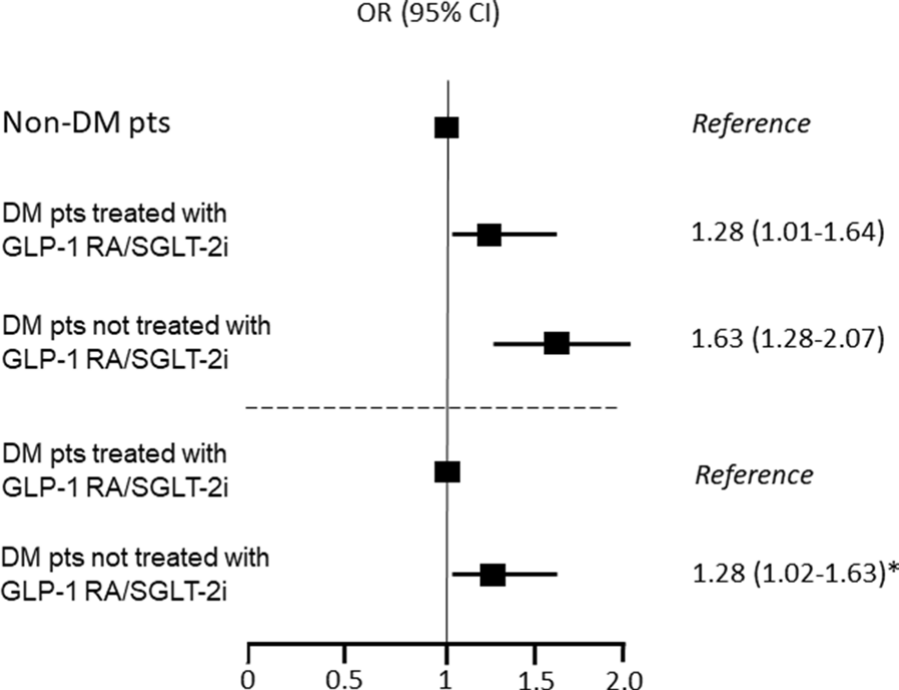
Primary endpoint risk in the study groups, after propensity score matching. Odds ratio and 95% confidence interval of the primary endpoint (composite of in-hospital mortality, acute heart failure, and acute kidney injury requiring renal replacement therapy) between the study groups, after propensity score matching. *OR was adjusted for chronic anti-hyperglycemic therapy. *CI* confidence interval, *DM* diabetes mellitus, *GLP-1 RA* glucagon-like peptide-1 receptor agonists, *OR* odds ratio, *SGLT-2i* sodium glucose cotransporter-2 inhibitors

Compared with non-DM patients, DM patients treated with GLP-1 RA/SGLT-2i had an almost 30% increased risk of the primary endpoint, while DM patients not treated with GLP-1 RA/SGLT-2i had an almost 60% increased risk (Fig. 3). When only patients with DM were considered, after adjustment for chronic anti-hyperglycemic therapy, those not treated with GLP-1 RA/SGLT-2i had a 30% higher risk of the primary endpoint than those treated with GLP-1 RA/SGLT-2i (Fig. 3). Incidence and risks of the secondary endpoints in the three study groups are reported in Table 3. A lower risk of each secondary endpoint was observed in DM patients treated with GLP-1 RA/SGLT-2i, when compared with DM patients not treated with these drugs.

**Table 3:** Rate and risk of the secondary endpoints in the three study groups after propensity score matching

	Non-DM pts (n = 1030)	DM pts treated with GLP-1 RA/SGLT-2i (n = 1030)	DM pts not treated with GLP-1 RA/SGLT-2i (n = 1030)	P value
In-hospital mortality				
n (%)	27 (3%)	35 (3%)	40 (4%)	0.10 (for trend)
OR (95% CI)	Reference	1.31 (0.78–2.18)	1.50 (0.91–2.46)	0.11 (for trend)
OR (95% CI)*	–	Reference	1.08 (0.67–1.76)	0.24
Acute heart failure				
n (%)	108 (10%)	137 (13%)	169 (16%)	< .0001 (for trend)
OR (95% CI)	Reference	1.31 (1.01–1.71)	1.68 (1.29–2.17)	< .0001 (for trend)
OR (95% CI)^a^	–	Reference	1.31 (1.02–1.70)	0.004
Severe AKI (RRT)				
n (%)	13 (1%)	3 (0.3%)	17 (2%)	0.39 (for trend)
OR (95% CI)	Reference	0.23 (0.07–0.81)	1.31 (0.63–2.72)	0.039 (for trend)
OR (95% CI)^a^	–	Reference	4.88 (1.36–17.6)	0.02

^a^Adjusted for anti-hyperglycemic therapy before hospitalization

*AKI* Acute kidney injury, *CI* confidence interval, *DM* diabetes mellitus, *OR* odds ratio, *RRT* renal replacement therapy

Among DM patients treated with GLP-1 RA/SGLT-2i, 48% of patients (n = 494) were on GLP-1 RA therapy at the time of admission, 50% (n = 516) were on SGLT-2i therapy and, finally, 2% (n = 20) were taking both drugs. Additional file 1: Table S1 reports the clinical characteristics of patients with DM treated with GLP-1 RA or SGLT-2i. A similar incidence of the primary endpoint was found between patients on GLP-1 RA and those on SGLT-2i (17% vs. 15%; P = 0.46, adjusted OR 1.34 [0.94–1.91]).

At the sensitivity analysis comparing DM patients treated with GLP-1 RA/SGLT-2i and those treated with DPP-4i, the former showed a significant adjusted lower risk of the primary endpoint (OR 0.45; 95% CI 0.37–0.55).

## Discussion

**Fig. 4:**
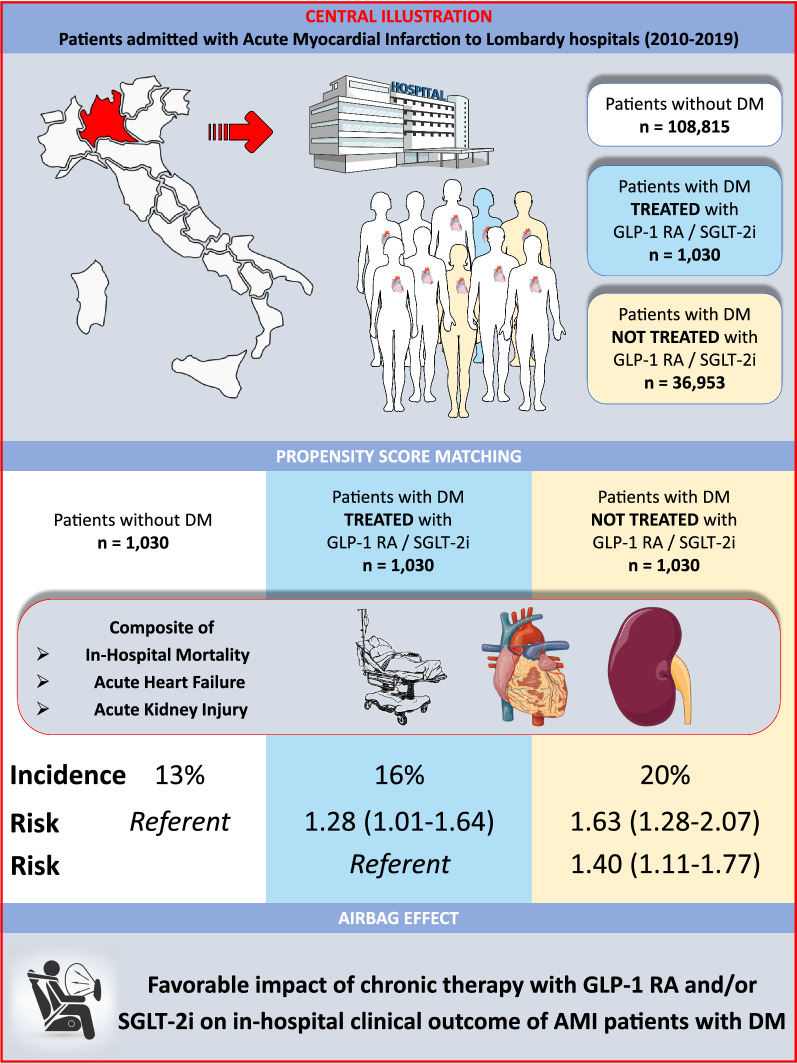
Summary of study design and main findings. Impact of chronic GLP-1 RA and/or SGLT-2i on in-hospital outcome of patients with diabetes mellitus hospitalized with acute myocardial infarction. *AMI* acute myocardial infarction, *CI* confidence interval, *DM* diabetes mellitus, *GLP-1 RA* glucagon-like peptide-1 receptor agonists, *OR* odds ratio, *SGLT-2i* sodium-glucose cotransporter-2 inhibitors

The results of the present study showed that patients with DM on chronic GLP-1 RA and/or SGLT-2i therapy have a better clinical outcome during hospitalization for AMI compared to those not treated with these anti-hyperglycemic drugs. In particular, the risk of in-hospital mortality, acute heart failure, and acute kidney injury requiring RRT in patients taking GLP-1 RA/SGLT-2i therapy was intermediate between that of patients without DM and that of DM patients not taking these drugs (Fig. 4).

Over the past decades, a significant improvement of outcomes in patients with AMI has been achieved, including in those with DM [22]. Despite this, worse in-hospital outcomes and a two-fold higher mortality have been consistently reported in DM patients [4, 23]. Their adverse prognosis has been traditionally associated with the presence of a higher burden of cardiovascular risk factors [24], a more diffuse and severe coronary atherosclerosis, less symptomatic AMI resulting in delayed hospital presentation, increased platelet activation and pro-coagulant state, and chronic inflammation [25]. More recently, it has been demonstrated that in AMI patients with DM the higher in-hospital mortality and major complication rates—including cardiogenic shock and acute kidney injury—are mainly driven by their more frequent cardiac and renal dysfunction at hospital presentation [8]. Thus, it can be speculated that preventive therapeutic interventions able to counteract acute cardio-renal injury during AMI may reduce the still existing mortality gap between DM and non-DM patients. In this regard, two classes of glucose-lowering agents—GLP-1 RA and SGLT-2i—are potential options to further reduce mortality in DM patients hospitalized with AMI. Indeed, data from large cardiovascular and renal outcome trials have highlighted that these drugs confer protection against major cardiac and renal events through mechanisms beyond their glucose-lowering effect. Most of these trials enrolled patients with established atherosclerotic cardiovascular disease, including those with a prior AMI, but only two trials were specifically focused on patients with a recent AMI. The ELIXA (Evaluation of LIXisenatide in Acute coronary syndromes) trial randomly assigned patients to receive lixisenatide (a GLP-1 RA) or placebo within 180 days from hospitalization for AMI (mean 72 days) [26]. In this trial, no difference in the primary composite endpoint (cardiovascular death, myocardial infarction, stroke, or unstable angina) rate and risk was found between the two arms at 3-year follow-up. The EMBODY study investigated the effects of empagliflozin, a SGLT-2i, initiated two weeks after AMI, in 105 patients with DM [27]. This trial reported that empagliflozin improved the cardiac sympathetic activity and prevented renal function decline at 6-month follow-up. Recent data from the SWEDEHEART registry confirmed that treatment with GLP-1 RA initiated soon after a first AMI in patients with DM is associated with a lower risk of major cardiovascular events at a median follow-up of 3 years [28]. To date, no study has evaluated the clinical effects of these two classes of drugs during the in-hospital phase of AMI.

To the best of our knowledge, our study is the first evaluating the potential impact of chronic GLP-1 RA and/or SGLT-2i therapy on in-hospital outcomes of DM patients hospitalized with AMI and treated with current standards of care. Indeed, although data collection of our study lasted 10 years, the matching procedure focused on patients in more recent years, mainly due to the progressively greater prescription of these drugs. Our analysis confirmed the close association between DM and worse in-hospital outcomes. However, when patients with DM were grouped according to their chronic anti-hyperglycemic therapy, those already treated with GLP-1 RA and/or SGLT-2i experienced a 30% lower risk of the primary endpoint than DM patients not on this therapy. The mechanisms underlying the clinical benefit of these drugs in the acute phase of AMI cannot be deduced from our data. However, we chose to combine acute heart failure and acute kidney injury requiring RRT with in-hospital mortality because of their strong prognostic impact on AMI, because they are less likely to be subject to coding error, and because of the well-known cardio- and renal-protective effects of these drugs. The finding that DM patients treated with GLP-1 RA and/or SGLT-2i showed a lower risk than those not treated with these drugs for each component of the primary endpoint supports a cardiac and renal protective effect of these drugs also during AMI hospitalization.

In line with our results, GLP-1 RA was demonstrated to improve myocardial function during AMI soon after successful percutaneous reperfusion and to reduce myocardial infarct size in both the clinical and preclinical settings [14, 15, 29]. In particular, Noyan-Ashraf et al. [30] showed, in an experimental model, that liraglutide administration for 7 days before AMI induction reduces infarct size and improves survival by engaging pro-survival pathways in cardiomyocyte. Moreover, chronic treatment with GLP-1 RA has been shown to reduce blood pressure and plasma levels of atherogenic lipoproteins, potentially resulting in a better hemodynamic profile and in a lower coronary atherosclerotic burden when AMI occurs [31–33]. Similarly, SGLT-2i administration was found to preserve cardiac contractile function and efficiency during myocardial ischemia–reperfusion injury, and also to improve metabolism and up-regulation of antioxidative proteins after coronary artery ligation in animal models [34]. Jiang et al. [35], using AMI mouse models with and without DM, demonstrated that treatment with a SGLT-2i significantly reduces infarct size and myocardial fibrosis, leading to improved cardiac function and survival. In the context of ischemia and nutritional glucose deprivation where autosis is already highly stimulated, SGLT-2i directly inhibits the activity of the Na + /H + exchanger 1 (NHE1) in the cardiomyocytes, significantly reducing autosis induced by glucose deprivation [35]. In contrast, overexpression of NHE1 aggravated the death response of cardiomyocytes to starvation, which was effectively reduced by SGLT-2i treatment. The analysis of NHE1, both in-vitro and in-vivo, confirmed that the cardioprotective effects of SGLT-2i occur, at least in part, via the downregulation of autophagy [35]. Finally, administration of SGLT-2i to patients with DM and a recent AMI was shown to reduce the progression of renal dysfunction [17]. It is also possible that dual SGLT-1 and SGLT-2 inhibition may give a clinical advantage, compared with GLP-1 RA and/or other SGLT-2i, in AMI patients. Indeed, it has been recently demonstrated that the relative increase in SGLT-1 inhibition with sotagliflozin is associated with a greater cardiovascular protection in patients with DM than the currently available SGLT-2i, that provide less degree of SGLT-1 inhibition [36].

Our findings, derived from administrative data, cannot be transferred directly into the clinical practice, but they do provide a strong rationale for further investigation on the potential clinical benefit of these agents also in the in-hospital treatment of AMI. Of note, information on ClinicalTrials.gov (updated to December 2022) shows that several randomized clinical trials focusing on the use of GLP-1 RA or SGLT-2i during AMI are currently ongoing. If the effects observed in the cardiovascular outcome studies are also reproduced in these trials, a sizeable proportion of patients hospitalized with AMI, and not only those with DM, will benefit from early treatment with these drugs [37]. Pending the results of these ongoing studies, our data suggest that in patients already on treatment with GLP-1 RA or SGLT-2i, these drugs should not be discontinued during AMI.

## Study limitations

Administrative databases are a reliable tool to describe outcomes of patient cohorts representing the real clinical care, since they collect data over time in a standardized way and at low cost. However, limitations that are typical of all the studies based on administrative datasets need to be acknowledged. Administrative data can suffer from systematic biases as their quality depends on the accuracy of coding. However, it should be highlighted that the endpoints considered in our study, as well as the variables chosen for risk adjustment, are less likely to be subject to coding error. Second, in our study, DM was defined according to chronic exposure to anti-hyperglycemic agents. Thus, patients with unknown DM and those treated with lifestyle and diet modification only were misclassified. Similarly, we were unable to distinguish between type 1 and type 2 DM. Third, the small number of events does not allow us to detect a potentially significant difference in the beneficial effect between GLP-1 RA and SGLT-2i or among single agents of the same class of drugs. Clinical trials of patients randomized to GLP-1 RA vs. SGLT-2i vs. both together are not currently available. However, one observational study found SGLT-2i to be more effective than GLP-1 RA in improving cardiovascular outcomes of DM patients [38] while another showed the superiority of GLP-1 RA [39], and a third one reported no differences in major cardiovascular events between these two drug classes [40]. In all these studies, the comparison was made in terms of prevention of cardiovascular events and not of protection during AMI, as in the present study. Fourth, GLP-1 RA and SGLT-2i can be prescribed in Italy only by diabetes specialists and this can represent a selection bias potentially identifying a population in which control of risk factors is more rigorous. However, at our sensitivity analysis comparing these drugs with DPP-4i, that similarly require a specialist prescription, the benefit observed with the former drugs was confirmed. Finally, some specific pieces of information on clinical variables or laboratory tests, including glycated hemoglobin, body mass index, estimated glomerular filtration rate, and left ventricular ejection fraction, which deserve attention when referring to outcomes in AMI, were not available. Similarly, information on withdrawal of oral diabetic medication during hospitalization for AMI could not be retrieved.

## Conclusions

Our study indicates that chronic therapy with GLP-1 RA and/or SGLT-2i has a favorable impact on the clinical outcome of DM patients hospitalized with AMI and seems to reduce the prognostic gap still existing between DM and non-DM patients. These promising data further support the rationale for the ongoing randomized clinical trials investigating these drugs in patients with AMI.

## Supplementary Information

**Additional file1**: **Table S1. **Clinical characteristics of DM patients treated with GLP-1 RA or SGLT-2i and hospitalized with acute myocardial infarction from 2010 to 2019.

## Data Availability

The data underlying this article were provided by the Lombardy region by permission and cannot be shared without permission of the Lombardy region.

## Abbreviations

AMI: Acute myocardial infarction
ATC: Therapeutic chemical classification
CI: Confidence intervals
DM: Diabetes mellitus
DPP-4i: Dipeptidyl peptidase 4 inhibitors
GLP-1 RA: Glucagon-like peptide-1 receptor agonists
OR: Odds ratio
SGLT-2i: Sodium glucose cotransporter-2 inhibitors
STEMI: ST-segment elevation myocardial infarction
