# Supplementary material for: Impact of chronic GLP-1 RA and SGLT-2I therapy on in-hospital outcome of diabetic patients with acute myocardial infarction
Source: Cardiovasc Diabetol. 2023 Feb 6;22:26. doi: 10.1186/s12933-023-01758-y (PMC9903538; doi:10.1186/s12933-023-01758-y)
Supplement: Supplementary file 1 — Additional file1: Table S1. Clinical characteristics of DM patients treated with GLP-1 RA or SGLT-2i and hospitalized with acute myocardial infarction from 2010 to 2019. [file 12933_2023_1758_MOESM1_ESM.docx]

**Supplementary Table S1.** Clinical characteristics of DM patients treated with GLP-1 RA or SGLT-2i and hospitalized with acute myocardial infarction from 2010 to 2019.

|  | **DM pts treated with GLP-1 RA**  (n=494) | **DM pts treated with**  **SGLT-2i**  (n=516) | *p value* |
| --- | --- | --- | --- |
| Age (years) | 67±9 | 66±9 | 0.30 |
| Female gender, n (%) | 139 (28%) | 135 (26%) | 0.48 |
| Year of hospitalization | 2017 (2014-2018) | 2018 (2017-2019) | <0.0001 |
| Prior MI, n (%) | 144 (29%) | 186 (36%) | 0.02 |
| STEMI as admission diagnosis, n (%) | 166 (34%) | 179 (35%) | 0.72 |
| ***Comorbidities^a^*** |  |  |  |
| Arterial hypertension, n (%) | 250 (51%) | 223 (43%) | 0.02 |
| Chronic IHD, n (%) | 215 (43%) | 263 (51%) | 0.02 |
| CKD, n (%) | 32 (6%) | 30(6%) | 0.66 |
| COPD, n (%) | 28 (6%) | 24 (5%) | 0.46 |
| Cancer, n (%) | 44 (9%) | 44 (8%) | 0.83 |
| Atrial fibrillation, n (%) | 26 (5%) | 30 (6%) | 0.70 |
| Chronic heart failure, n (%) | 4 (1%) | 9 (2%) | 0.19 |
| Number of comorbidities |  |  | 0.10 |
| 0, n (%) | 454 (92%) | 446 (86%) |  |
| 1, n (%) | 32 (6%) | 56 (11%) |  |
| 2, n (%) | 5 (1%) | 8 (2%) |  |
| 3, n (%) | 1 (0.2%) | 4 (11%) |  |
| >3, n (%) | 2 (0.4%) | 2 (0.4%) |  |
| ***Medications before hospitalization*** |  |  |  |
| ACE-I/ARBs, n (%) | 386 (78%) | 373 (72%) | 0.03 |
| Anti-hypertensive drugs, n (%) | 436 (88%) | 458 (89%) | 0.80 |
| Beta-blockers, n (%) | 280 (57%) | 305 (59%) | 0.43 |
| Lipid lowering drugs, n (%) | 391 (79%) | 418 (81%) | 0.46 |
| Antiplatelet drugs, n (%) | 300 (61%) | 315 (61%) | 0.92 |
| Anticoagulant drugs, n (%) | 32 (6%) | 35 (7%) | 0.84 |
| ***Anti-hyperglycemic drugs*** |  |  |  |
| Metformin, n (%) | 407 (82%) | 412 (80%) | 0.30 |
| Sulfonylurea, n (%) | 209 (42%) | 82 (16%) | <0.0001 |
| DPP-4i, n (%) | 47 (10%) | 43 (8%) | 0.51 |
| Insulin, n (%) | 160 (32%) | 297 (58%) | <0.0001 |
| ***Procedures during index hospitalization*** |  |  |  |
| Coronary multivessel disease, n (%) | 353 (71%) | 379 (73%) | 0.48 |
| PCI, n (%) | 344 (70%) | 375 (73%) | 0.28 |
| DES implantation, n (%) | 310 (63%) | 338 (65%) | 0.36 |
| ***Endpoints*** |  |  |  |
| Primary endpoint, n (%) | 84 (17%) | 79 (15%) | 0.46 |
| In-hospital mortality, n (%) | 17 (3%) | 18 (3%) | 0.97 |
| Acute heart failure, n (%) | 70 (14%) | 64 (12%) | 0.41 |
| AKI requiring RRT, n (%) | 2 (0.4%) | 1 (0.2%) | 0.54 |

Abbreviations: ACE-I = angiotensin-converting-enzyme inhibitors; AKI = acute kidney injury; ARBs = angiotensin II receptor blockers; CKD = chronic kidney disease; COPD = chronic obstructive pulmonary disease; DES = drug-eluting stent; DM = diabetes mellitus; DPP-4i = dipeptidyl peptidase-4 inhibitors; IHD = ischemic heart disease; MI = myocardial infarction; PCI = percutaneous coronary intervention; RRT = renal replacement therapy; STEMI = ST-elevation myocardial infarction.

20 patients treated with both classes of drugs were excluded from the analysis.

^a^in the previous 10 years.
